# Multiple pathways are responsible for Anti-inflammatory and Cardiovascular activities of *Hordeum vulgare* L.

**DOI:** 10.1186/s12967-014-0316-9

**Published:** 2014-11-26

**Authors:** Saima Gul, Sagheer Ahmed, Nurolaini Kifli, Qazi Tahir Uddin, Nafisa Batool Tahir, Abrar Hussain, Hawa ZE Jaafar, Marius Moga, Muhammad Zia-Ul-Haq

**Affiliations:** PAPRSB Institute of Health Sciences, University Brunei Darussalam, Gadong BE1410 Bander Seri Begawan, Brunei Darussalam; Department of Pharmacy, Kohat University of Science & Technology, Kohat, 26000 Pakistan; Department of Surgery, Khyber Medical University Institute of Medical Sciences, Kohat, 26000 Pakistan; Department of Medicine, Khyber Medical University Institute of Medical Sciences, Kohat, 26000 Pakistan; Department of Biotechnology & Informtics, Baluchistan University of Information Technology, Engineering & Management Sciences, Quetta-87300 Quetta, Pakistan; Department of Crop Science, Faculty of Agriculture, University Putra Malaysia, 43400 Selangor, Malaysia; Department of Medical and Surgical Specialities, Transilvania University of Brasov, Brasov, Romania; The Patent Office, Karachi, Pakistan

**Keywords:** Inflammation, *Hordeum vulgare*, Platelets, Cyclooxygenase, Lipoxygenase, Superoxide dismutase, Glutathione peroxidase

## Abstract

**Background:**

*Hordeum vulgare* L. (HV or barley) is used by traditional healers to treat various inflammatory and cardiovascular diseases, without the knowledge of pharmacologic rationale behind its actions. This study was designed to explore the potential scientific mechanism(s) that could explain the use of *Hordeum vulgare* in traditional medicine as a treatment for various inflammatory and cardiovascular diseases.

**Methods:**

A crude extract and its three fractions were prepared from HV and screened for the inhibition of platelet aggregation and various metabolites of cyclooxygenase (COX), lipoxygenase (LOX) pathways of arachidonic acid (AA) metabolism as well as for its effects on certain antioxidant enzymes. Platelet aggregation was monitored using turbidometric principle, AA metabolism through radioimmunoassay and antioxidant enzymes by commercial kits using spectrophotometer.

**Results:**

Results show that HV exhibited activities against all human platelet agonists used except adenine diphosphate, and inhibited both COX and LOX pathways of AA metabolism. It also elevated the activities of superoxide dismutase (SOD) and glutathione peroxidase (GPx). However, these activities were distributed in various fractions of HV. Aqueous fraction was most potent in elevating SOD activity; chloroform fraction had concentrated compounds responsible for COX inhibition while n-hexane seems to possess compounds responsible for LOX inhibition as well as the only fraction enhancing the activity of GPx.

**Conclusions:**

These results suggest the likely mechanisms responsible for observed anti-inflammatory and cardiovascular effects of HV in traditional medicine.

## Background

World literature, especially, Greek, Roman and Egyptian literatures point to the dietary and medicinal properties of *Hordeum vulgare* L. (HV) [1]. HV, a member of the grass family, *Poaceae,* is an annual crop used throughout ancient civilizations as an important food source. The genus *Hordeum* consists of 32 species and 45 taxa. HV belong to a single biological species, which is an annual and is diploid. All varieties of HV have hollow stems in the forms of cane, produced from fibrous roots. There is a spike-shaped arrangement of seed at the end of each stem.

Until the 19^th^ century, barley was the major part of diet of millions of people. Even now, it is eaten as such or in processed form, in various quantities at various ontogenic stages by people of all income and age groups globally as functional food. Various clinical and pre-clinical studies have confirmed the positive and beneficial effects of barley on human health. Consumption of barley which contains many medicinally active phytocompounds is usually associated with improvement in health. Use of barley can protect against diabetes, obesity, atherosclerosis, cancer, stroke and insulin resistance [2].

A number of pharmacological activities of HV have been reported in the literature. Hot water extracts of HV protect from damage due to oxidation of various proteins [3]. Anti-apoptotic activity of the HV has been reported and is attributed to the presence of 3,4 dehydroxybenzaldehyde, which protects from DNA damage [4]. Lipopolysaccharide-induced inflammation is also inhibited by methanolic extract of the aerial parts of HV, both *in vitro* and *in vivo* [5]. Barley or HV is also used to treat a number of diseases in traditional Ayurvedic system of medicine in India, especially in the treatment of urinary stones [6]. The growth of the urinary stones [7] as well as mild hyperuricemia [8] was found to be inhibited by using the seed extracts of HV.

The main constituents of HV include important antioxidants such as vitamin E, phytic acid, selenium, tocotrienols, and various phenolic acids. After the consumption of HV, these antioxidants are released at differential rate throughout the gastrointestinal tract over a long period of time [9]. Health benefits of HV consumption are numerous [10] and are mainly attributed to various mineral, dietary fibres, unsaturated fatty acids and sphingolipids, in addition to the antioxidants and vitamins [11]. Apart from that a number of other antioxidant compounds have been isolated from HV [12].

HV is used by traditional healers to treat various inflammatory and cardiovascular diseases, without the knowledge of pharmacologic rationale behind its actions [6]. Despite the very important roles of platelets activation in the development of acute thrombosis and cardiovascular diseases (CVD), no data are available concerning the effect of barley on platelet activation thrombus formation. Since a number of plants have been reported to display inhibition of arachidonic acid (AA) metabolism and platelet aggregation [13-18], we therefore, screened various fraction of HV against cyclooxygenase (COX) and lipoxygenase (LOX) pathways of AA metabolism. Since both of these enzymatic pathways are implicated in oxidative stress, we further evaluated the actions of HV on superoxide dismutase (SOD) and glutathione peroxidase (GPx). These activities have not been previously reported for HV.

## Experimental section

The chemicals used in the study were purchased from BDH (acetic acid and citric acid), Merck (sodium phosphate-mono and dibasic), Amersham Biosciences (^14^C arachidonic acid) and Sigma (the remaining), all from USA and were of analytical grade.

### Extraction of plant material

Rotary evaporator was used to make crude extract of the 2000 gof barley seeds which was authenticated by a botanist and a specimen kept at the department of Pharmacy, Kohat University of Science & Technology, Kohat, Pakistan. Barley seeds were crushed to powder before soaking it in the solvent. Five liter of aquous methanol was used to soak 1500 g powdered bark at 4°C and left for 24 h. This mixture was filtered and then evaporated to obtain the crude extract by a rotary evaporator [19]. The resulting extract was a solid mass of dark brown color.

### Fractionation of the crude extract

Fractionation of the crude extract was done by slight modification of the method described previously [20]. Crude extract (900 g) was dissolved in distilled water before intoroducing into a separating funnel. After addition of 50 ml of n-hexane, the whole mixture was vigorously mixed and allowed to stand at the room temperature for 30 min. This resulted in separation of two layes. After acquiring the upper layer of n-hexane, this frcationation process was repeated twice and the resulting three n-hexane fractions were combined and evaporated in the rotary evaporator to obtain n-hexane fraction. Next, 50 ml chloroform was added to the remaining layer and again the whole fractionation process was repeated as with n-hexane fraction, thus obtaining chloroform fraction. After that, the remaining layer was concentrated in the rotary evaporated to get aquous fraction.

### Arachidonic acid metabolism by human platelets

Formation of AA metabolites by human platelets and its inhibition by various fractions of barley was measurd by slight modification of a method described previously [21]. Concentarted plasma (50 ml) was obtained from the diagnostic laboratories of the Aga Khan University, Karachi. Platelet rich plasma (PRP) was obtained by subjecting concentrated platelets to centrifugation for 20 min at 1200 g. This PRP was treated with phosphate buffer twice before resuspending them in the same buffer without EDTA.

The suspension was centrifuged for 20 min at 1200 g after homogenizing the PRP suspension for 15 seconds at 4°C using a polytron homogenizer. A 300 μl supernatant was incubated with 10 μg unlabelled AA and 0.1 μCi [1-^14^C]AA in the presence and absence of various HV fractions. The reaction was allowed to proceed at 37°C for 15 min. Citric acid and ethyle acetate was used to stop the reaction. The organic layer was separated under recommended standard conditions and then dried. The residues were dissolved and applied on thin layer chromatography plates. Standards for AA, thromboxane (TX) B_2_ (a stable degradation product of TXA_2_), lipoxygenase product-1 (LP1) and 12-hidroxy eicosatetraenoic acid (12-HETE) were plotted separately [22]. The plates were developed using different solvent systems for different products. Quantification with a Berthold TLC linear analyzer and chromatography data system was done after locating the radioactive zones.

### Preparation of platelets

Nine ml blood was obtained from healthy human volunteers who were not on any medication for at least last 10 days. One ml of sodium citrate (3.8%, w/v) was added to the blood and mixed gently. This sample was centifuged at lower speed (at 260 g) for 15 min to get platelet rich plasma (PRP). To get the platelet poor plasma (PPP), the remaining blood was centrifuged at higher speed (at 1200 g) for 10 min. The centrifugation was done at 20°C while the actual aggregation studies were performed at 37°C. Platelet count of PRP was between 2.5 and 3.0 × 10^8^ ml^–1^ of plasma [23] as determined by phase contrast microscopy.

### Measurement of platelet aggregation

Platelet aggregation was monitored in 450 μl of PRP [24,25]. The final volume of PRP was made upto 500 μl by the addition of test sample and aggregation agonist. Platelet were challenged with four aggregating agonits; platelet activating factor (PAF) (0.8 μM), AA (1.7 mM), Adenine diphosphate (ADP) (2.2 μM), and collagen (5 μg/ml). Platelets were incubated with test fraction for 2 min before the addition of aggregating agonist. After challenging platelets with agonist, results were recorded for 5 min as measured by a change in the transmission of light as a function of time. Dose response curves were calculated for various fractions against different agonists and used to calculate the 1C_50_ values.

### Glutathione peroxidase activity determination

GPx activity was measured as described previously [26]. The test fraction is added to assay solution followed by the addition of the substrate (t-butyl-hydroperoxide) which is the last component of assay added before the chemical reaction starts. This reaction works by bringing together the reduced form of nicotinamide adenine dinucleotide phosphate (NADPH) and reduced glutathione. Addition of the substrate starts its reduction and a change in the absorbance is measured at 340 nm. GPx activity is finally determined using glutathione as co-substrate.

### Superoxide dismutase activity determination

SOD activity was measured as described previously [27]. As done in GPx assay, the test fraction is added to assay solution followed by the addition of the substrate which is the last component of assay added before the chemical reaction starts. In this assay the substrate, 2-(4-iodophenyl)- 3-(4-nitrophenol)-5-phenyltetrazolium chloride (INT)) is converted to a formazane dye. Superoxide radicals are produced by xanthine and xanthine oxidase. SOD activity is directly related to the extent of inhibition of this reaction and is measured by detecting a change in absorbance at 505 nm. Under such conditions, 50% inhibition rate is equivalent to one unit of SOD activity.

### Statistical analysis

The doses of the extract and fractions showing 50% effect (IC_50_ or EC_50_) were calculated. Means between the groups were compared using student’s t-test. The difference between the means was considered significant when p <0.05.

## Results

**Figure 1:**
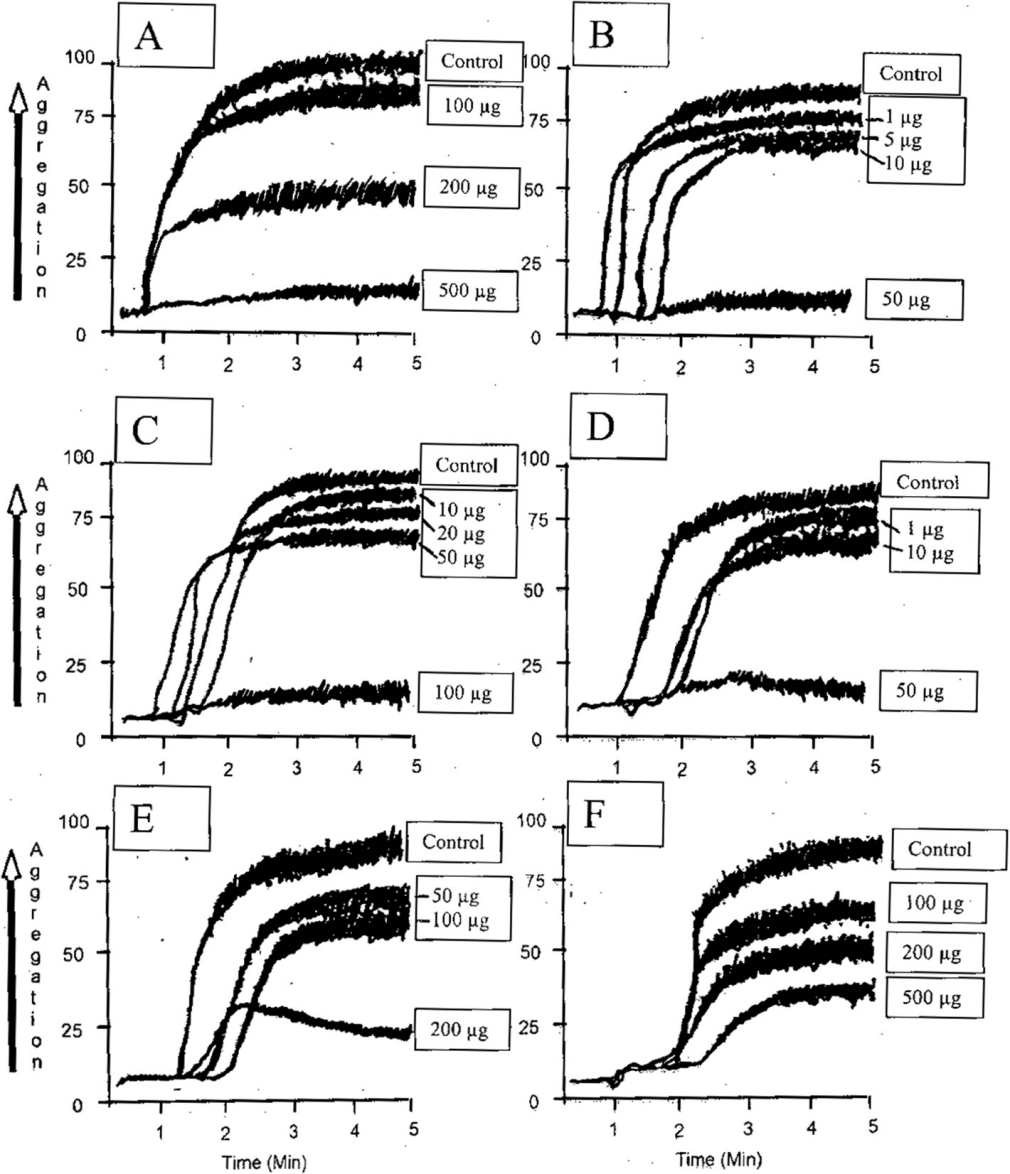
**Representative scans of the effect of crude extract of HV and its fractions on platelet aggregation induced by various agonists, i.e. crude on PAF (A), crude on AA (B), aqueous on AA (C), chlororform on AA (D), n-hexane on PAF (E), aqueous on collagen (F).**

Crude extract from HV was unable to inhibit ADP-induced platelet aggregation; however, collagen and PAF-induced aggregations were blocked partially while the most potent antiplatelet effect was observed against AA-induced-platelet aggregation. The dose of the extract showing 50% inhibitory effect against platelet aggregation (mean IC_50_ value in μg) against various agonists were 18.33 ± 3.51 for AA, 187.71 ± 19.57 for PAF, 211.23 ± 27.92 for collagen and 15.88 ± 2.14 for aspirin (positive standard control) (Table 1 and Figure 1). Crude extract of HV was also more effective against COX-mediated pathway of AA metabolism compared to LOX-mediated pathway. IC_50_ (mean ± SD in μg) values were 24.34 ± 5.33 for TXB_2_, 78.45 ± 12.29 for LP1 and 89.44 ± 16.22 for HETE. Crude extract potently elevated SOD activity (EC_50_ in μg, 32.88 ± 3.19) but was ineffective in increasing GPX activity up to 1 mg dose (Table 2).

**Table 1:** **IC**_**50**_ **(mean (SD)) of the crude extract of HV and its fractions against various platelet agonists, AA metabolites and antioxidant enzymes**

**No**	**Experiment**	**Crude (μg)**	**Aqueous (μg)**	**Chloroform (μg)**	**n-Hexane (μg)**
**1**	PAF	187.71 (19.57)	1mg	98.56 (16.68)	97.82 (15.63)
**2**	AA	18.33 (3.51)	58.73 (12.82)	21.63 (4.10)	86.74 (12.54)
**3**	Collagen	211.23 (27.92)	121.55 (20.32)	126. 73 (22.36)	1mg
**4**	ADP	1mg	1mg	1mg	1mg
**5**	LP-1	78.45 (12.29)	1mg	66.52 (10.51)	37.61 (4.71)
**6**	12-HETE	89.44 (16.22)	1mg	118.44 (19.75)	60.52 (4.60)
**7**	TXB_2_	24.34 (5.33)	74.67 (12.52)	28.63 (6.76)	40.34 (7.46)
**8**	SOD (EC_50_)	32.88 (3.19)	28.21 (4.73)	28.83 (5.48)	35.29 (5.33)
**9**	GPx (EC_50_)	1mg	1mg	1mg	23.17 (3.29)

**Table 2:** Activities of SOD and GPx under normal and stressed conditions and the effects of the crude fraction of HV and its fractions, n = 6, *p <0.05 compared to normal

	**Dose (μg)**	**SOD activity**	**Dose (μg)**	**GPx activity**
**Normal**	-	171 ± 9	-	5872 ± 642
**Vitamin C (Standard)**	10	215 ± 20*	10	8751 ± 761*
**H**_**2**_ **O**_**2**_ **(Stress)**	0.3 μM	148 ± 13*	0.3 μM	4192 ± 523*
**Crude**	20	184 ± 21	100	6145 ± 765
	50	198 ± 19	500	6171 ± 625
	100	212 ± 24*	1000	6211 ± 566
**Aqueous**	10	181 ± 18	100	6257 ± 621
	30	189 ± 20	500	6304 ± 724
	50	198 ± 17*	1000	6326 ± 844
**Chloroform**	10	179 ± 16	100	6352 ± 628
	30	194 ± 20	500	6386 ± 863
	50	207 ± 21*	1000	6456 ± 767
**n-hexane**	20	184 ± 21	10	6002 ± 590
	50	197 ± 23	20	7109 ± 653*
	100	213 ± 20*	50	8663 ± 759*

Aqueous fraction of HV was unable to inhibit PAF and ADP-induced platelet aggregation, however, AA and collagen -induced aggregations were blocked partially. The dose of the fraction showing 50% inhibitory effect against platelet aggregation (IC_50_ value in μg) against various agonists were 58.73 ± 12.82 for AA, 121.55 ± 20.32 for PAF (Table 1 and Figure 1). Aqueous fraction of HV was ineffective against LOX-mediated pathway of AA metabolism while partially blocking COX-mediated pathway. IC_50_ (mean ± SD in μg) values were 74.67 ± 12.52 for TXB_2_, while no significant effect was observed on LP-1 and 12-HETE up to a dose of 1 mg. Aqueous fraction just like the crude extract potently elevated SOD activity (EC_50_ in μg, 28.21 ± 4.73) but was ineffective in increasing GPX activity up to 1 mg dose (Table 2).

**Figure 2:**
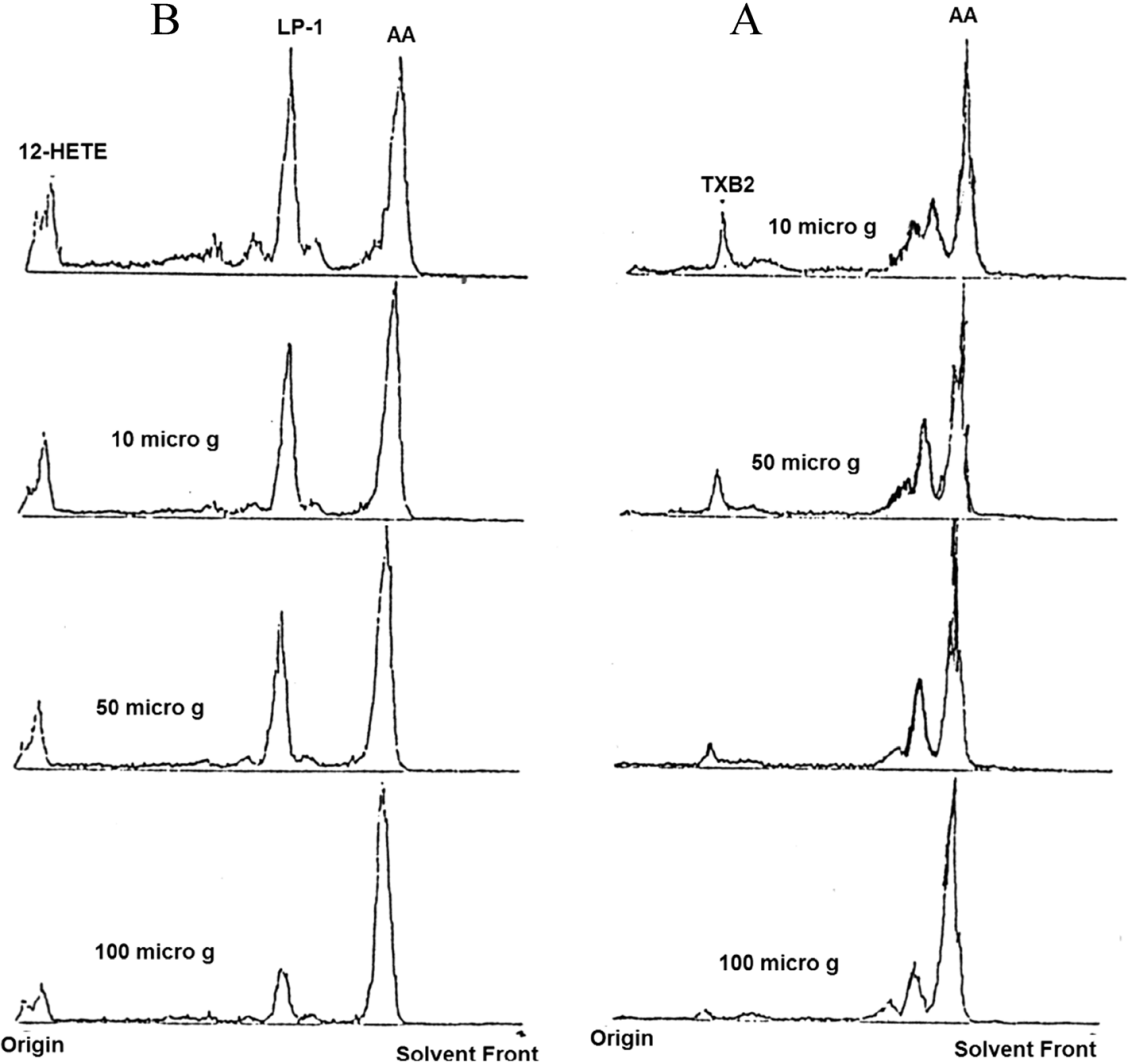
**A** **representative scan of the effect of n-hexane fraction of HV on AA metabolism through COX (A) and LOX (B) enzymatic pathways.**

Chloroform fraction of HV, just like the crude extract, was unable to inhibit ADP-induced platelet aggregation; however, collagen and PAF-induced aggregations were blocked partially while the most potent antiplatelet effect was observed against AA-induced-platelet aggregation. The dose of the fraction showing 50% inhibitory effect against platelet aggregation (IC_50_ value in μg) against various agonists were 21.63 ± 4.10 for AA, 98.56 ± 16.68 for PAF, 126.73 ± 22.36 for collagen (Table 1 and Figure 1). Chloroform fraction of HV was also more effective against COX-mediated pathway of AA metabolism compared to LOX-mediated pathway. IC_50_ (mean ± SD in μg) values were 28.63 ± 6.76 for TXB_2_, 66.52 ± 10.51 for LP1 and 118.44 ± 19.75 for HETE. Chloroform fraction just like the crude extract and aqueous fraction potently elevated SOD activity (EC_50_ in μg, 28.83 ± 5.48) but was ineffective in increasing GPX activity up to 1 mg dose (Table 2).

n-hexane fraction of HV was unable to inhibit collagen and ADP-induced platelet aggregation, however, AA and PAF-induced aggregations were blocked partially. The dose of the fraction showing 50% inhibitory effect against platelet aggregation (IC_50_ value in μg) against various agonists were 86.74 ± 12.54 for AA, 97.82 ± 15.63 for PAF (Table 1 and Figure 1). LOX pathway of AA metabolism was blocked potently while COX metabolite was partially inhibited. IC_50_ (mean ± SD in μg) values were 40.34 ± 7.46 for TXB_2_, 37.61 ± 4.71 for LP1 and 60.52 ± 4.60) for HETE (Figure 2). n-hexane potently elevated the activities of both SOD and GPx with EC_50_ (μg) of 35.29 ± 5.33 and 23.17 ± 3.29 respectively (Table 2).

## Discussion

Despite cardiovascular protective potential of available synthetic drugs, these drugs have various side-effects like skin rashes, bleeding, headaches and gastrointestinal problems. Therefore research is now directed towards green pharmaceuticals (medicines of plant origin) which are believed to be free from side-effects. Plants have been used for centuries to obtain treatment for various human diseases. One of the most important therapeutic targets for treating cardiovascular and inflammatory disease is the inhibition of AA metabolites through COX [28-30]. There are reports suggesting natural inhibition of AA metabolites [31]. Dual inhibition of LOX and COX pathways by natural compounds is also known and thought to be contributed by the secondary metabolism of plants [32,33]. Natural inhibitors of both inflammation and oxidative stress, however, are less common but could be of great therapeutic importance. Here in this report, we show that HV strengthens the antioxidant defense system and inhibits LOX and COX pathways of AA metabolism.

In the present investigation, crude extract of HV showed potent inhibition of AA-induced platelet aggregation, was partially effective against PAF and collagen while no significant effect was observed against ADP. These results suggest that crude fraction inhibits platelet aggregation mainly acting through COX pathway as AA induces platelet aggregation after conversion to TXA2 by platelet COX enzyme. Inhibition of thromboxane-mediated platelet aggregation, however, seems to be a common mechanism shared by many other plants [3,15-17,34]. Further experiments with AA metabolism also suggest that crude fraction blocks COX mediated pathway of AA as it significantly inhibited TXB2 production from AA while both metabolites of LOX pathway were only partially inhibited. Crude extract was devoid of any significant effect on GPx, however, SOD activities were strongly elevated by the application of crude extract.

Both COX and LOX enzymatic pathways are implicated in oxidative stress and studies indicate that inhibition of COX [26] as well as LOX [35,36] reduces oxidative stress. Our results show that crude extract of HV as well as all three fractions elevated SOD activity while only n-hexane fraction enhances GPx activity. Previous studies also suggest that inhibition of COX lowers the oxidative stress [37]. Rise in SOD activity are likely to be a consequence of COX inhibition which can up regulates antioxidant enzymes such as SOD [38]. Similarly COX-2 and GPx are inversely related [39] and the effect of HV in this case may be related to its ability to inhibit COX. A number of studies show the presence of antioxidant potential of medicinal plants [32,33] including increasing activities of SOD and GPx [26].

Aqueous fraction of HV did not show any significant activity against platelet aggregation with only partially inhibiting AA and collagen while having no significant effect on PAF and ADP. Interestingly aqueous fraction had no effect on LOX mediated metabolism of AA while only partial inhibition of TXB_2_ was observed suggesting aqueous fraction has less of compounds responsible for AA metabolism. This result also explains relatively low effect of aqueous fraction on AA-induced platelet aggregation. Just like crude extract, aqueous fraction showed strong elevation of SOD activity but ineffective against GPx. Consistent with previous studies [40,41], these results suggest that aqueous fraction has concentrated compounds that have potent effect on SOD and that the fractionation process has resulted in the distribution of compounds responsible for COX inhibition into other fractions as well.

Chloroform fraction of HV showed activities similar to the crude extract and akin to previous reports [42,43], strongly inhibited TXB_2_ production and AA-induced platelet aggregation and having significant effect on elevating SOD activities. This suggests that chloroform fraction retains the activities contained in the crude extract and that fractionation process did not affect much in the distribution of phytocompounds in chloroform fraction. This also means that SOD elevation activity of HV is probably exhibited by more than one compound because aqueous fraction also had similar effects on SOD. Chloroform fraction of other plants has been shown previously to possess activities against platelet aggregation [44].

The pattern of activities observed with n-hexane fraction of HV was slightly different and exhibited strong effect against LOX-mediated metabolites of AA and significantly elevated GPx activity. These two activities are not shared by any other fraction, nor by the crude extract. Similar activities were previously found in n-hexane fraction of other plants [45,46]. This suggests that crude extract might contain ingredients that do not allow compounds responsible for these activities to show any significant effect and act as their blockers.

Metabolism of AA, especially by two enzymatic pathways-LOX and COX produces various eicosanoids which play crucial role in inflammation [47]. Therefore, all the diseases with underlying inflammation are likely to benefit from inhibition of AA metabolism [48]. Since inflammation is associated with weakening antioxidant defense, the results of our study show that HV that possesses phytocompounds with anti-inflammatory as well as anti-oxidant potential can be used in such circumstances. Our study signifies the importance of HV as a starting point for the development of novel compounds against two of the most important enzymes implicated in inflammation in addition to enhancing activities of SOD and GPx. It is possible that separate compounds are responsible for activities related to COX, LOX, SOD and GPx; however, further, studies may also reveal that two or more of the above activities are possessed by a single phytocompound. Further characterization of HV and its fractions and isolation of pure compounds could provide us valuable information in this context.

## Conclusions

Our study demonstrates that HV possesses activities against all human platelet agonists used in this study except ADP, and inhibited both COX and LOX pathways of AA metabolism. It also elevated the activities of SOD and GPx. However, these activities were distributed in various fractions. Therefore, it is unlikely that a single phytocompound is responsible for all these activities. Some fractions of HV showed more potent activities than others while certain fractions were almost completely devoid of anti-inflammatory effect (aqueous). Interestingly, crude extract was unable to enhance GPx activity while n-hexane fraction which was obtained from the fractionation of crude extract, caused significant elevation of GPx activities.

## References

[1] Wood R (1988). The Whole Foods Encyclopedia.

[2] Behall KM, Scholfield DJ, Hallfrisch J (2004). Diets containing barley significantly reduce lipids in mildly hypercholesterolemic men and women. Am J Clin Nutr.

[3] Duh P, Yen G, Yen W, Chang L (2001). Antioxidant effect of water extracts from barley prepared under different roasting temperatures. J Agric Food Chem.

[4] Jeong JB, Hong SC, Jeong HJ (2009). 3,4-Dihydroxybenzaldehyde purified from the barley seeds (*Hordeum vulgare*) inhibits oxidative DNA damage and apoptosis via its antioxidant activity. Phytomed.

[5] Choi KC, Hwang JM, Bang SJ, Son YO, Kim BT, Kim DH, Lee SA, Chae M, Kim H, Lee JC (2013). Methanol extract of the aerial parts of barley (*Hordeum vulgare*) suppresses lipopolysaccharide-induced inflammatory responses *in vitro* and *in vivo*. Pharm Biol.

[6] Khare CP (2007). Indian Medicinal Plants-An Illustrated Dictionary.

[7] Shah JG, Patel BG, Patel SB, Patel RK (2012). Antiurolithiatic and antioxidant activity of *Hordeum vulgare* seeds on ethylene glycol-induced urolithiasis in rats. Indian J Pharmacol.

[8] Hokazono H, Omori T, Yamamoto T, Akaoka I, Ono K (2010). Effects of a fermented barley extract on subjects with slightly high serum uric acid or mild hyperuricemia. Biosci Biotechnol Biochem.

[9] Tsai CJ, Leitzmann MF, Willett WC, Giovannucci EL (2004). Long-term intake of dietary fiber and decreased risk of cholecystectomy in women. Am J Gastroenterol.

[10] Goldberg RJ, Ciampa J, Lessard D (2007). Longterm survival after heart failure, a contemporary population-based perspective. Arch Intl Med.

[11] Murtaugh MA, Jacobs DR, Jacob B (2003). Epidemiological support for the protection of whole grains against diabetes. J Proc Nut Soc.

[12] Etoh H, Murakami K, Yogoh T, Ishikawa H, Fukuyama Y, Tanaka H (2004). Anti-oxidative compounds in barley tea. Biosci Biotechnol Biochem.

[13] Zia-Ul-Haq M, Shahid SA, Ahmed S, Ahmad S, Qayum M, Khan IU (2012). Anti-platelet activity of methanolic extract of *Grewia asiatica* L. leaves and *Terminalla chebula* Retz. fruits. J Med Plants Res.

[14] Zia-ul-Haq M, Khan BA, Landa P, Kutil Z, Ahmed S, Qayum M, Ahmad S (2012). Platelet aggregation and anti-inflammatory effects of garden pea, desi chickpea and kabuli chickpea. Acta Poloniae Pharma.

[15] Zia-Ul-Haq M, Ahmed S, Rizwani GH, Qayum M, Ahmad S, Hanif M (2012). Platelet aggregation inhibition activity of selected legumes of Pakistan. Pak J Pharm Sci.

[16] Hussain J, Jamila N, Gilani SA, Abbas G, Ahmed S (2009). Anti-Platelet aggregation, antiglycation, cytotoxic, phytotoxic and antimicrobial activities of extracts of *Nepeta juncea*. African J Biotech.

[17] Hussain J, Khan FU, Gilani SA, Abbas G, Ahmed S, Khan AU, Choudhary MI (2010). Antiglycation, antiplatelets aggregation, cytotoxic and phytotoxic activities of *Nepeta suavis*. Latin Amer J Pharm.

[18] Imran I, Hussain L, Ahmed S, Rasool N, Rasool S, Abbas G, Ali MY (2012). Antiplatelet activity of methanolic extract of *Acacia leucophloea* bark. J Med Plants Res.

[19] Ahmed S, Gul S, Gul H, Bangash MH (2013). Anti-inflammatory and anti-platelet activities of *Avena sativa* are mediated through the inhibition of cyclooxygenase and lipoxygenase enzymes. Int J Endorsing Health Sci.

[20] Ahmed S, Gul S, Gul H, Bangash MH (2013). Dual inhibitory activities of Adhatoda vasica against cyclooxygenase and lipoxygenase. Int J Endorsing Health Sci.

[21] Aslam R, Saeed SA, Ahmed S, Connor JD (2008). Plasma lipoproteins inhibit platelet aggregation and arachidonic acid metabolism in experimental hypercholesterolemia. Clin Exp Pharmacol Physiol.

[22] Saeed SA, Connor JD, Quadri J, Tasneem S, Ahmed S, Mesaik MA, Choudhary MI (2007). Inhibitors of phosphatidylinositide 3-kinase: effects on reactive oxygen species and platelet aggregation. Pharmacol Rep.

[23] Saeed SA, Ahmad N, Ahmed S (2007). Dual inhibition of cyclooxygenase and lipoxygenase by human haptoglobin: its polymorphism and relation to hemoglobin binding. Biochem Biophys Res Commun.

[24] Saeed SA, Motiwala A, Qureshi ZU, Khan R, Ali A, Quadri J, Ahmed S (2006). Mechanisms of platelet aggregation mediated by G-protein coupled receptors in human platelets. J Coll Physicians Surg Pak.

[25] Ahmed S, Gul S, Zia-Ul-Haq M, Stanković MS (2014). Pharmacological basis of the use of *Acorus calamus* L. in inflammatory diseases and underlying signal transduction pathways. Latin Amer Caribbean Bull Med Aromatic Plant.

[26] Gul S, Ahmed S, Gul H, Kaneez KF (2011). Investigating the protective effect of *Solanum melongena*. Asian J Health.

[27] Gul S, Ahmed S, Gul H, Shad KF, Zia-Ul-Haq M, Badiu D (2013). The antioxidant potential of *Brassica rapa* L. on glutathione peroxidase, superoxide dismutase enzymes and total antioxidant status. Revista Română de Medicină de Laborator.

[28] Saeed SA, Khan SK, Ahmed S (2003). The inhibition of prostaglandin biosynthesis by human plasma and its relationship to albumin and haptoglobin. J Bio Sci.

[29] Saeed SA, Ahmed S, Ali A (2003). A New Function of Human Haptoglobin: Endogenous inhibition of prostaglandin biosynthesis and its relation to hemoglobin binding. J Medical Sci.

[30] Saeed SA, Rasheed H, Ali TH, Ahmed S (2004). Mechanisms of inhibitory actions of cyclooxygenase-2 inhibitors in human platelets. J Bio Sci.

[31] Saeed SA, Ahmed S (2005). New aspects of cyclooxygenase-2 inhibition in myocardial infarction and ischaemia. Res Comm Molecul Pathol Pharmacol.

[32] Saeed SA, Ahmed S (2006). Anti-ischemic effects of nimesulide, a cyclooxygenase-2 inhibitor on the ischemic model of rabbit induced by isoproterenol. Arch Pharm Res.

[33] Saeed SA, Ahmed S (2006). Role of cyclooxygenase-2 in myocardial infarction and ischemia. J Coll Physicians Surg Pak.

[34] Bukhari IA, Khan RA, Gilani AH, Ahmed S, Saeed SA (2010). Analgesic, anti-inflammatory and anti-platelet activities of the methanolic extract of *Acacia modesta* leaves. Inflammopharmacology.

[35] Lee JA, Song HY, Ju SM, Lee SJ, Seo WY, Sin DH, Goh AR, Choi SY, Park J (2010). Suppression of inducible nitric oxide synthase and cyclooxygenase-2 by cell-permeable superoxide dismutase in lipopolysaccharide-stimulated BV-2 microglial cells. Mol Cells.

[36] Chinnici CM, Yao Y, Ding T, Funk CD, Praticò D (2005). Absence of 12/15 lipoxygenase reduces brain oxidative stress in apolipoprotein E-deficient mice. Am J Pathol.

[37] Czapski GA, Czubowicz K, Strosznajder RP (2012). Evaluation of the antioxidative properties of lipoxygenase inhibitors. Pharm Rep.

[38] Kwiecien S, Konturek PC, Sliwowski Z, Mitis-Musiol M, Pawlik MW, Brzozowski B, Jasnos K, Magierowski M, Konturek SJ, Brzozowski T (2012). Interaction between selective cyclooxygenase inhibitors and capsaicin-sensitive afferent sensory nerves in pathogenesis of stress-induced gastric lesions. Role of oxidative stress. J Physiol Pharmacol.

[39] Liao X, Wang L, Yang C, He J, Wang X, Guo R, Lan A, Dong X, Yang Z, Wang H, Feng J, Ma H (2011). Cyclooxygenase mediates cardioprotection of angiotensin-(1-7) against ischemia/reperfusion-induced injury through the inhibition of oxidative stress. Mol Med Rep.

[40] Banning A, Kipp A, Schmitmeier S, Löwinger M, Florian S, Krehl S, Thalmann S, Thierbach R, Steinberg P, Brigelius-Flohé R (2008). Glutathione peroxidase 2 inhibits cyclooxygenase-2-mediated migration and invasion of ht-29 adenocarcinoma cells but supports their growth as tumors in nude mice. Cancer Res.

[41] Ahmed S, Gul S, Gul H, Zia-Ul-Haq M, Iram S, Jaafar HZ, Moga M (2014). Scientific basis for the use of *Cinnamonum tamala* in cardiovascular and inflammatory diseases. Exp Clin Card.

[42] Ahmed S, Gul S, Gul H, Shad KF, Zia-Ul-Haq M, Ercisli S, Jaafar HZ, Moga M (2014). Clinical justification of ethnomedicinal use of *Brassica rapa* in cardiovascular diseases. Exp Clin Card.

[43] Zhao WX, Jin MH, Li T, Wang YJ, Quan JS (2013). Intervention effect of aqueous fractions from *Boschniakia rossica* on hepatic oxidative stress in mice with liver injury induced by carbon tetrachloride. Zhongguo Zhong Yao Za Zhi.

[44] Son DJ, Lim Y, Park YH, Chang SK, Yun YP, Hong JT, Takeoka GR, Lee KG, Lee SE, Kim MR, Kim JH, Park BS (2006). Inhibitory effects of *Tabebuia impetiginosa* inner bark extract on platelet aggregation and vascular smooth muscle cell proliferation through suppressions of arachidonic acid liberation and ERK1/2 MAPK activation. J Ethnopharmacol.

[45] Thiombiano AME, Adama H, Jean BM, Bayala B, Nabèrè O, Samson G (2014). In vitro antioxidant, lipoxygenase and xanthine oxidase inhibitory activity of fractions and macerate from *Pandiaka angustifolia* (Vahl) Hepper. J Applied Pharm Sci.

[46] Konaté K, Souza A, Coulibaly AY, Meda NT, Kiendrebeogo M, Lamien-Meda A, Millogo-Rasolodimby J, Lamidi M, Nacoulma OG (2010). In vitro antioxidant, lipoxygenase and xanthine oxidase inhibitory activities of fractions from *Cienfuegosia digitata* Cav., *Sida alba* L. and *Sida acuta* Burn f. (Malvaceae). Pak J Biol Sci.

[47] Ahmed S, Gul S, Zia-Ul-Haq M, Riaz M: **Hypolipedemic effects of nimesulide and celecoxib in experimentally-induced hypercholesterolemia in rabbits.** *Turk J Med Sci*. In Press. 10.3906/sag-1312-106 26084115

[48] Ahmed S, Gul S, Gul H, Zia-Ul-Haq M (2014). Cyclooxygenase-2 inhibition improves anti-oxidative defence during experimental hypercholesterolemia. Bosn J Basic Med Sci.

